# Methylene blue for the diagnosis of a sinus tract in periprosthetic knee joint infection

**DOI:** 10.5194/jbji-6-423-2021

**Published:** 2021-11-16

**Authors:** Simon Martin Heinrich, Parham Sendi, Martin Clauss

**Affiliations:** 1 Centre for Musculoskeletal Infections, University Hospital Basel, Spitalstrasse 21, Basel 4031, Switzerland; 2 Department of Orthopaedic and Trauma Surgery, University Hospital Basel, Basel, Switzerland; 3 Department of Infectious Diseases and Hospital Epidemiology, University Hospital Basel, Basel, Switzerland; 4 Institute for Infectious Diseases, University of Bern, Bern, Switzerland

An 83-year-old woman presented to our clinic with a persistent wound drainage after removal of a baker's cyst located in her right knee. She underwent total knee arthroplasty (TKA) 6 months prior and had several weeks of discomfort in the popliteal region. Magnetic resonance imaging of the knee showed a large baker's cyst with a slightly enhanced synovial signal in the knee. Open removal of the baker's cyst was performed. The wound did not heal after surgery, and a revision was performed 12 d later. Biopsy samples were obtained, and empiric antibiotic treatment with amoxicillin/clavulanate was started. *Serratia marcescens* grew in three out of three biopsy samples, and antibiotic therapy was streamlined to intravenous cefepime. A total of 7 d later, treatment was switched to oral ciprofloxacin. The patient was then referred to our institution due to persistent compromised wound healing in the setting of presumed superficial infection, which possibly required a muscle flap.

On physical examination, there were no local signs of inflammation, though watery fluid secretion from the popliteal wound was observed. Periprosthetic joint infection (PJI) was diagnosed based on the presence of the draining sinus near the prosthetic implant. Diagnostic arthrocentesis with an arthrography (methylene blue and contrast medium injection into the knee) was performed. The dispersion of the radiopaque substance under fluoroscopy is illustrated in Fig. 1a. The next day, the leakage of methylene blue through the popliteal wound was observed (Fig. 1b and c), proving the communication to the knee joint.

**Figure 1:**
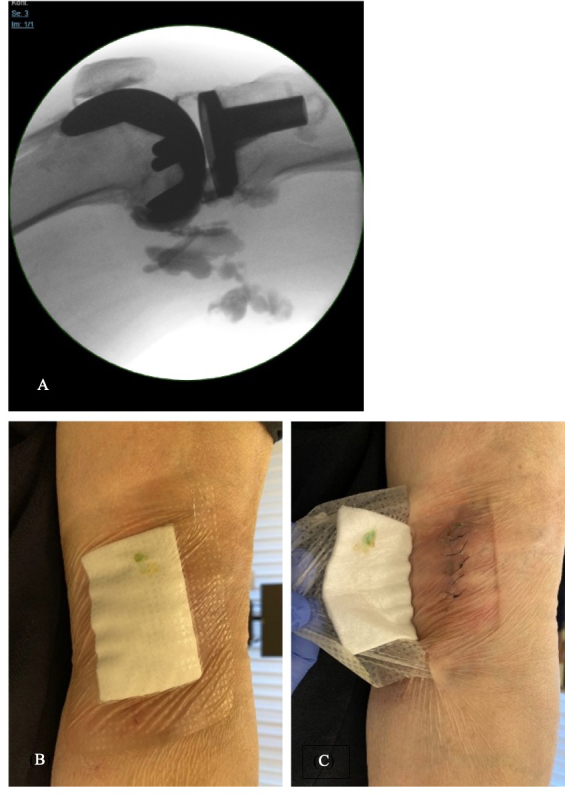
**(a)** Dispersion of the contrast medium under fluoroscopy. **(b, c)** Leakage of methylene blue through the popliteal wound. © University Hospital of Basel.

Surgical site infection can be categorized as being superficial, deep and organ/space (National Healthcare Safety Network, 2021). However, this categorization is inappropriate when there is an implant in place. A superficial wound infection over a foreign body is an implant-associated infection until proven otherwise (Berbari et al., 1998). The use of intra-articular methylene blue dye is an old fashioned, but still useful, method to confirm the extent of a sinus tract (Ziv et al., 2009).

## Data Availability

No data sets were used in this article.
